# Ret-dependent and Ret-independent mechanisms of Gfl-induced sensitization

**DOI:** 10.1186/1744-8069-7-22

**Published:** 2011-03-30

**Authors:** Brian S Schmutzler, Shannon Roy, Sherry K Pittman, Rena M Meadows, Cynthia M Hingtgen

**Affiliations:** 1 Department of Pharmacology and Toxicology, Indiana University School of Medicine, 635 Barnhill Drive, Indianapolis, Indiana, 46202, USA; 2 Department of Neurology, Indiana University School of Medicine, 545 Barnhill Drive, Emerson Hall, Room 125, Indianapolis, Indiana, 46202, USA; 3 Program in Medical Neurobiology, Indiana University School of Medicine, 950 West Walnut Street, R2, Room 402, Indianapolis, Indiana, 46202, USA

## Abstract

**Background:**

The GDNF family ligands (GFLs) are regulators of neurogenic inflammation and pain. We have previously shown that GFLs increase the release of the sensory neuron neuropeptide, calcitonin gene-related peptide (CGRP) from isolated mouse DRG.

**Results:**

Inhibitors of the mitogen-activated protein kinase (MAPK) pathway abolished the enhancement of CGRP release by GDNF. Neurturin-induced enhancement in the stimulated release of CGRP, used as an indication of sensory neuronal sensitization, was abolished by inhibition of the phosphatidylinositol-3 kinase (PI-3K) pathway. Reduction in Ret expression abolished the GDNF-induced sensitization, but did not fully inhibit the increase in stimulus-evoked release of CGRP caused by neurturin or artemin, indicating the presence of Ret-independent GFL-induced signaling in sensory neurons. Integrin β-1 and NCAM are involved in a component of Ret-independent GFL signaling in sensory neurons.

**Conclusions:**

These data demonstrate the distinct and variable Ret-dependent and Ret-independent signaling mechanisms by which GFLs induce sensitization of sensory neurons. Additionally, there is a clear disconnect between intracellular signaling pathway activation and changes in sensory neuronal function.

## Background

The glial cell line-derived neurotrophic factor (GDNF) family ligands (GFLs) are a group of small peptides in the TGFβ super-family of molecules. They exist naturally as homodimers and include GDNF, neurturin (NRTN), artemin (ARTN), and persephin [PSPN; 1, 2]. There is direct evidence that the GFLs can alter channel properties and the threshold of activation of sensory neurons. Interestingly, application of GDNF, NRTN, and ARTN enhance calcium influx through TRPV1 in sensory neurons exposed to capsaicin, a specific exogenous ligand for the channel [3]. We have demonstrated that the change in sensitivity of sensory neurons elicited by GDNF, NRTN, and ARTN results in increased release of the neuropeptide, calcitonin gene-related peptide [CGRP; 4], an important transmitter in neurogenic inflammation and pain signaling.

Each of the GFLs has its own GDNF family receptor α (GFRα) subtype to which it preferentially binds. The action of the GFRα receptors, which are localized to lipid rafts by the GPI-anchors [5], is initiated when a GFL homodimer approaches two GFRα receptors, of the same isoform, and causes them to homodimerize [6]. This GFL-GFRα complex translocates to the Ret receptor tyrosine kinase and causes a dimerization of Ret, which initiates a number of intracellular signaling pathways [6]. The intracellular signaling pathways initiated by Ret are diverse, including MEK-Erk 1/2 [6,7], phospatidylinositol-3 kinase (PI-3K) [8,9], Jun NH2-terminal protein kinase [10], p38 MAPK [11], and phospholipase C-gamma [PLC-γ; 12]. There is evidence that activation with different GFLs results in distinct Ret confirmations and initiation of unique signaling cascades [13]. In addition, there is emerging evidence of GDNF-induced, Ret-independent signaling through Src family kinases (SFKs), and the MEK-Erk 1/2 and pCREB pathways [5]. Neural cell adhesion molecules (NCAMs) were the first alternative GFRα-1 binding partners identified [14,15], but GFRα-1 can bind with Integrin β1 as well [16]. Although there is no functional evidence of other Ret-independent GFL-mediated actions, these data suggest the possibility of Ret-independent signaling in other neurons.

Here we demonstrate that each of the GFLs uses distinct intracellular signaling pathways to elicit sensory neuronal sensitization, measured by enhancement in the capsaicin stimulated-release of the sensory neuron neuropeptide, CGRP. We are able to distinguish activation of signaling pathways by the individual GFLs from the pathways involved in sensory neuronal sensitization. Additionally, we identify Ret-independent signaling pathways initiated by NRTN and ARTN, which are important in altering the function of peripheral sensory neurons. These complements of signaling pathways necessary for GFL-induced inflammation and pain signaling are novel.

## Results and Discussion

### Ret-independent signaling pathways are responsible for NRTN and ARTN-induced enhancement in the release of iCGRP

**Figure 1:**
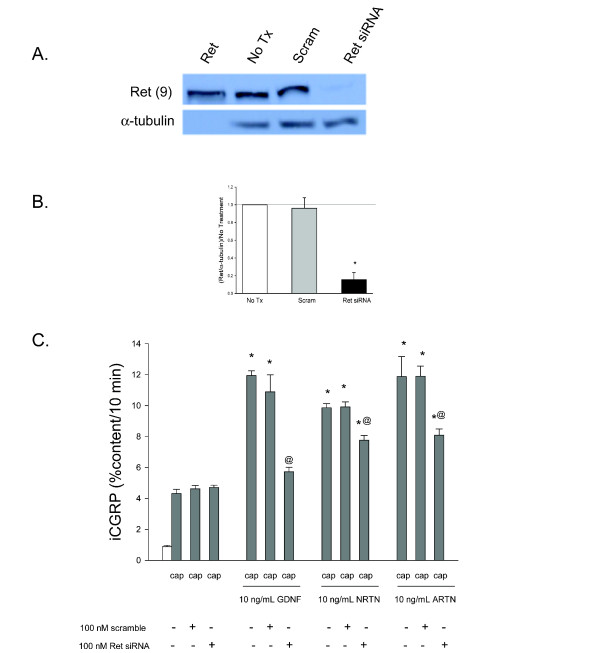
**GDNF-induced enhancement in the stimulated-release of iCGRP is mediated by Ret-dependent pathways**. A) This representative Western blot demonstrates that exposure of DRG to Ret siRNA decreases Ret levels, while scramble siRNA does not change Ret levels. Additionally, a portion of the Ret protein, 31-330, is present on the immunoblot with a band the same size as Ret from DRG. B) Densitometric analysis of three separate Western blots like that in A probing for Ret. C) Peptide release elicited by a 10 minute exposure to Hepes buffer alone (open bar) or Hepes buffer containing 50 nM capsaicin (Cap; dark bars) is expressed as mean percent total peptide content of cells in each well ± SEM (n = 12-18 wells per condition). Asterisks (*) indicate statistically significant differences in band density and iCGRP release between treatment groups and the no GFL condition using an ANOVA with Dunnett's post-hoc test (p < 0.05). Ampersands (@) indicate statistically significant differences between the GFL treatment and the siRNA treated condition using a t-test (p < 0.05). In all cases, release stimulated by capsaicin was significantly higher than basal release.

Several studies indicate a Ret-independent component to GFLs' actions [14-17], although these studies provide only indirect evidence of Ret-independent function. To determine if Ret is necessary for the GFL-induced sensitization of primary sensory neurons involved in neurogenic inflammation and pain modulation, the ability of GFLs to enhance capsaicin-stimulated release of immunoreactive CGRP (iCGRP) in isolated mouse sensory neurons with reduction in the expression of Ret was examined. Capsaicin activates the TRPV1 receptor expressed on peptide containing sensory neurons that mostly fall into the category of small diameter nociceptive neurons [18,19]. Our DRG preparation is a heterogeneous compilation of several different types of neurons and glial cells. Almost all neurons that express the TRPV1 receptor also express CGRP [20,3]. In addition, each of the specific GFRα subtypes is coexpressed with TRPV1 at varying levels, with approximately 20-50% coexpression of GFRα-1 or GFRα-2 with TRPV1 and a nearly 90% coexpression of GFRα-3 and TRPV1 [3,21,22]. Our previous work demonstrates that GDNF, NRTN and ARTN all enhance capsaicin-induced release of iCGRP from these cultures, indicating the co-expression of the three GFRα subtypes with TRPV1 in significant subsets of peptide containing neurons in our preparation [4]. In these cultures of dorsal root ganglia (DRG) neurons, Ret levels were reduced with the use of a specific pool of siRNA molecules directed at Ret, since no specific pharmacological inhibitors of the activation of this molecule exist. The siRNA pool (100 nM) reduced the amount of Ret protein in the DRG cultures by ~85%, while a scramble siRNA did not alter Ret levels (Figure 1A and B). As shown in Figure 1, the immunoband present in the lane loaded with the purified Ret protein is at same location as the bands for the DRG tissue probed with Ret antibody.

As we have previously demonstrated, exposure of DRG cultures to GDNF, ARTN or NRTN (at 10 ng/ml for 20 min) causes an approximate 2 fold increase in capsaicin-stimulated release of iCGRP (Figure 1C). These treatments do not alter resting levels of iCGRP release or the total neuronal content of the peptide [4]. To determine whether Ret is necessary for GFL-induced enhancement of iCGRP release, Ret siRNA (100 nM) was added to the DRG in culture two days after plating and remained in the culture media for 48 hours. Interestingly, while the GDNF-induced sensitization of sensory neurons was abolished when Ret siRNA was added, NRTN and ARTN still elicited an increase in stimulated peptide release (Figure 1C). The enhancement in stimulated-evoked release of iCGRP by NRTN and ARTN, while still present, was significantly reduced in the presence of Ret siRNA. The total content of iCGRP was not affected by these manipulations. Therefore, Ret is necessary for the enhanced stimulated-release of iCGRP induced by GDNF and mediates a component of the enhancement caused by NRTN and ARTN. The observation that NRTN and ARTN are still capable of enhancing the stimulated-release of CGRP in sensory neurons in cultures with greatly reduced Ret expression, suggests that a component of the enhancement caused by NRTN and ARTN is due to Ret-independent mechanisms.

**Figure 2:**
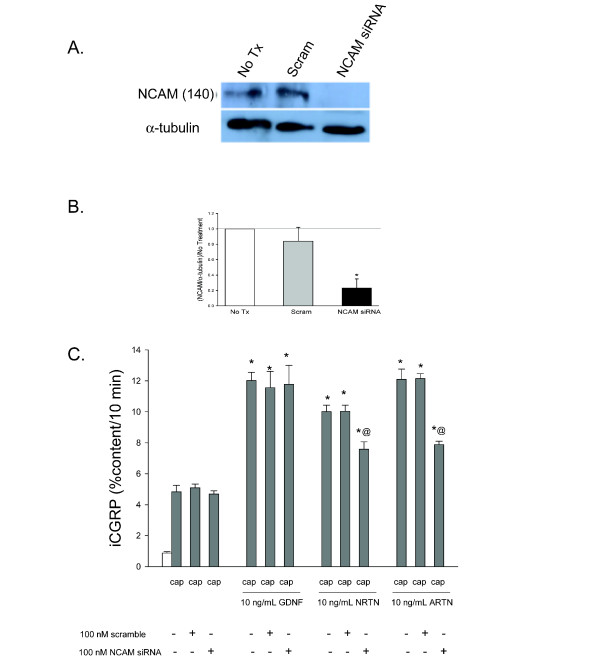
**ARTN-induced enhancement in the stimulated-release of iCGRP is mediated, in part, by NCAM-dependent pathways**. A) This representative Western blot demonstrates that exposure of DRG to NCAM siRNA decreases NCAM levels, while scramble siRNA does not change NCAM levels. B) Densitometric analysis of three separate Western blots like that in A probing for NCAM. C) Peptide release elicited by a 10 minute exposure to Hepes buffer alone (open bar) or Hepes buffer containing 50 nM capsaicin (Cap; dark bars) is expressed as mean percent total peptide content of cells in each well ± SEM (n = 12-18 wells per condition). GFLs were included in the 10 minutes prior to and throughout capsaicin exposure. Asterisks (*) indicate statistically significant differences in band density and iCGRP release between treatment groups and the no GFL condition using an ANOVA with Dunnett's post-hoc test (p < 0.05). Ampersands (@) indicate statistically significant differences between the GFL treatment and the siRNA treated condition using a t-test (p < 0.05).

One of the other possible binding partners of the GFL-GFRα complexes is NCAM [15]. To investigate whether or not NCAM is involved in the NRTN and ARTN-induced sensitization of DRG neurons, NCAM levels were decreased using a pool of siRNA directed towards NCAM p140. NCAM p140 is the intracellular portion of this protein responsible for initiation of intracellular signaling pathways, specifically the Fyn kinase pathway [23]. The pool of NCAM siRNA reduced NCAM p140 levels by ~75% in DRG cultures (Figure 2A and 2B). The amount of NCAM present in untreated and scramble siRNA treated DRG cultures were not different. Additionally, reduction of the level of NCAM by NCAM siRNA in the DRG cultures did not prevent the GDNF-induced enhancement in the stimulated-release of CGRP (Figure 2C).

**Figure 3:**
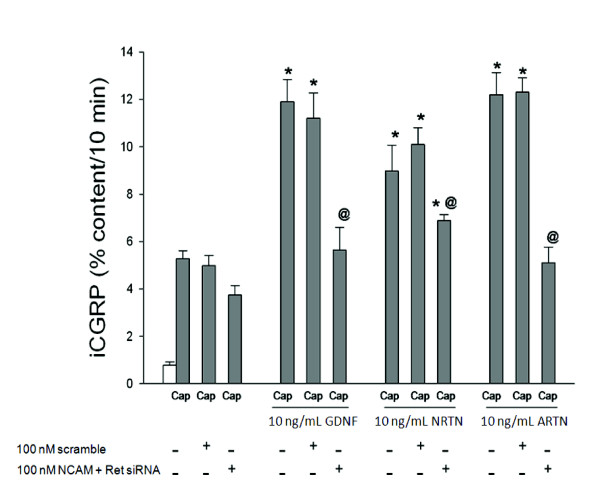
**ARTN-induced enhancement in the stimulated-release of iCGRP is mediated by Ret-dependent and NCAM-dependent pathways**. Peptide release elicited by a 10 minute exposure to Hepes buffer alone (open bar) or Hepes buffer containing 50 nM capsaicin (Cap; dark bars) is expressed as mean percent total peptide content of cells in each well ± SEM (n = 12-18 wells per condition). Asterisks (*) indicate statistically significant differences in iCGRP release between treatment groups and the no GFL condition using an ANOVA with Dunnett's post-hoc test (p < 0.05). Ampersands (@) indicate statistically significant differences between the GFL treatment and the siRNA treated condition using a t-test (p < 0.05). In all cases, release stimulated by capsaicin was significantly higher than basal release.

DRG cultures were treated NCAM siRNA (50 nM) and Ret siRNA (50 nM), to ensure the total amount of siRNA present in the culture media was consistent (100 nM) and the basal and stimulated-release of iCGRP was measured in the presence of GFLs. Figure 3 shows that GDNF-induced sensitization was abolished with this combined siRNA treatment regimen, presumably because the enhancement in stimulated-release of iCGRP accomplished by GDNF is Ret-dependent, as seen in Figure 1. The ARTN-induced sensitization, while not eliminated by Ret siRNA treatment alone, was completely abolished by NCAM and Ret siRNA treatment in combination (10 ng/mL ARTN: 12.13 ± 0.91%,10 ng/mL ARTN and Ret + NCAM siRNA: 5.22 ± 0.61%). NRTN-induced sensitization was not prevented by NCAM and Ret siRNA treatment in combination. However, the enhancement of stimulated release of iCGRP in response to NRTN was significantly lower with the combined siRNA treatment (10 ng/mL NRTN: 9.09 ± 1.27%,10 ng/mL NRTN and Ret + NCAM siRNA: 7.20 ± 0.37%). Treatment with NCAM siRNA alone did not abolish NRTN or ARTN-induced sensitization, although these treatments did reduced the amount of GFL-induced release, and NCAM siRNA treatment alone did not affect GDNF-induced enhancement in the stimulated-release of CGRP (Figure 2C).

**Figure 4:**
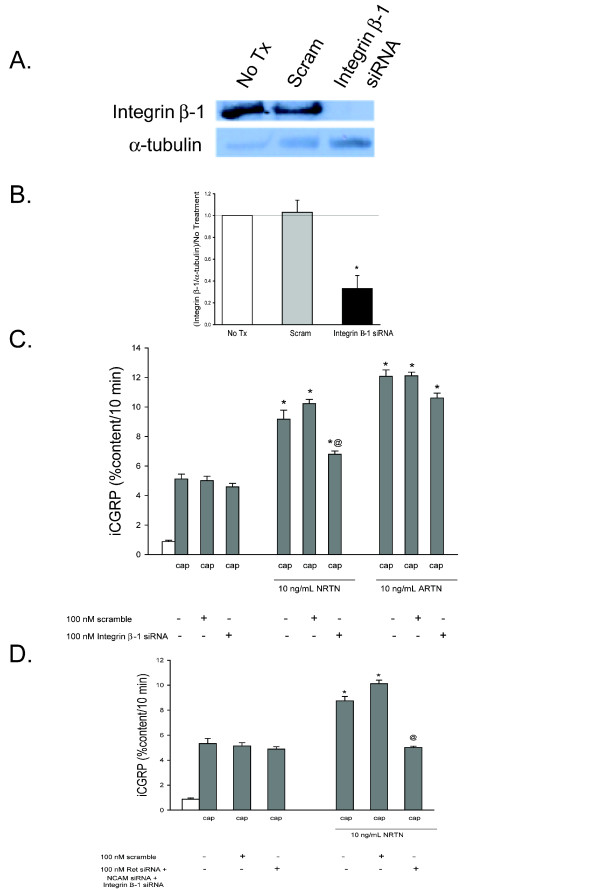
**NRTN-induced enhancement in the stimulated-release of iCGRP is mediated by Ret-dependent, NCAM-dependent, and Integrin β-1-dependent pathways**. A) This representative Western blot demonstrates that exposure of DRG to Integrin β-1 siRNA decreases Integrin β-1 levels, while scramble siRNA does not change Integrin β-1 levels. B) Densitometric analysis of three separate Western blots like that in A probing for Integrin β-1. C and D) Peptide release elicited by a 10 minute exposure to Hepes buffer alone (open bar) or Hepes buffer containing 50 nM capsaicin (Cap; dark bars) is expressed as mean percent total peptide content of cells in each well ± SEM (n = 12-18 wells per condition). Asterisks (*) indicate statistically significant differences in band density and iCGRP release between treatment groups and the no GFL condition using an ANOVA with Dunnett's post-hoc test (p < 0.05). Ampersands (@) indicate statistically significant differences between the GFL treatment and the siRNA treated condition using a t-test (p < 0.05). In all cases, release stimulated by capsaicin was significantly higher than basal release.

Finally, the role of another receptor reported to be a binding partner of the GFL-GFRα complex, Integrin β-1, was investigated [16]. A pool of siRNA molecules (100 nM) directed at Integrin β-1 was used in order to inhibit its expression and this was verified with a Western blot probing for the Integrin β-1 intracellular fragment, which has a molecular weight of 130 kDa and is the direct signaling portion of the molecule [24]. Figure 4A and 4B demonstrate that Integrin β-1 siRNA reduces the level of expression of this receptor by ~65% in DRG cultures.

When Integrin β-1 siRNA was added to DRG, NRTN-induced sensitization remained, although the level of enhancement in stimulated release of iCGRP was reduced (Figure 4C). Integrin β-1 siRNA did not affect the ARTN-induced enhancement in the stimulated-release of CGRP. The total content of iCGRP was not affected by these manipulations. This data indicates that Integrin β-1 plays a role in NRTN-induced sensitization, but that it is not the only mechanism by which NRTN induces sensitization.

DRG cultures were then treated with Ret siRNA, NCAM siRNA, and Integrin β-1 siRNA and exposed to the GFLs to determine the role of these complements of receptors in GFL-induced sensitization. DRG were treated with all three siRNA (33 nM each) on day 2, 4, and 6 after plating. This treatment regimen was followed to ensure the total amount of siRNA present in the culture media was consistent (100 nM) and that over the course of the three treatments the cells were exposed to 100 nM of each siRNA. When all three pools of siRNA were added to the DRG in culture, the basal-release of iCGRP was not affected while the NRTN-induced sensitization of stimulus-evoked release was abolished (Figure 4D). The total content of iCGRP was not affected by these manipulations. These data indicate that all three of these receptors (Ret, NCAM, and Integrin β-1) are important in NRTN-induced enhancement in the stimulated-release of CGRP. There is a Ret-dependent component, which contributes about half of the NRTN-induced enhancement in the capsaicin stimulated-release of iCGRP. Two Ret-independent pathways, NCAM-dependent and Integrin β-1-dependent pathways, account for the other components of the NRTN-induced enhancement in the capsaicin stimulated-release of iCGRP. However, it is necessary to inhibit all three of these pathways to eliminate NRTN-induced sensitization, which is a novel observation for the mechanism of the GFL-induced actions on sensory neurons. There are two possible scenarios to explain this phenomenon. Either the GFRα-2 containing neurons that respond to NRTN contain all three signaling receptors (Ret, NCAM and Integrin β-1), each of which play a role in the sensitizing action of NRTN on that individual neuron or, alternatively, there are different populations of GFRα-2 expressing neurons expressing only one or two of the signaling receptors. For example, when siRNA to Ret is used this eliminates the response from neurons containing GFRα-2 and Ret but the activity of other neurons expressing GFRα-2 and NCAM and/or Integrin β-1 is preserved.

### Distinct signaling pathways are responsible for GFL-induced enhancement in the release of iCGRP

Having established that selected GFLs induce sensitization of sensory neurons through different complements of cell surface receptors, the intracellular signaling pathways by which the GFLs induce sensitization were determined. Most of the evidence for the actions of the GFLs on adult, mammalian neurons indicates that the following signaling pathways are activated by GFL-induced activation of Ret: MEK/Erk 1/2 pathway, the PI-3K pathway, the Src kinase pathway, the Fyn pathway, and the PKC pathway [25-28]. These same intracellular signaling pathways are initiated by NCAM activation [15,29] and Integrin β-1 activation [30].

**Figure 5:**
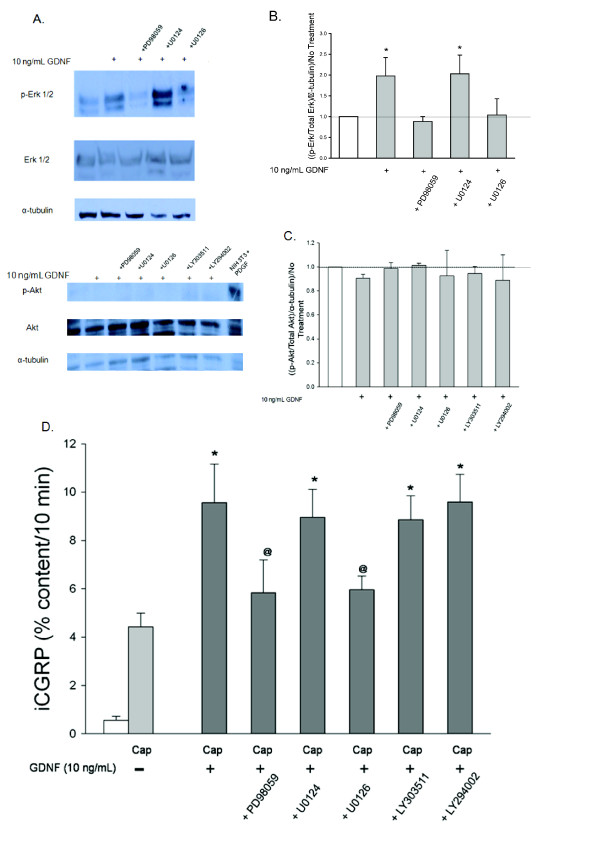
**GDNF-activates the MAPK pathway and not the PI-3K pathway and sensitizes sensory neurons through the MAPK pathway**. A) This representative Western blot demonstrates changes in levels of p-Erk 1/2, Erk 1/2, p-Akt, Akt, and α-tubulin in DRG in response to a 10 minute incubation with 10 ng/mL GDNF and the subsequent prevention of these changes by inhibitors of the MAPK pathway (10 μM PD98059 and 1 μM U0126) and the PI-3K pathway (10 μM LY294002). B and C) Densitometric analysis of three separate Western blots like that in A probing for B) MAPK pathway components and C) PI-3K pathway components. D) Peptide release elicited by a 10 minute exposure to Hepes buffer alone (open bar) or Hepes buffer containing 50 nM capsaicin (Cap; dark bars) is expressed as mean percent total peptide content of cells in each well ± SEM (n = 12-18 wells per condition). GDNF and inhibitors.were included in the 10 minutes prior to and throughout capsaicin exposure. Asterisks (*) indicate statistically significant differences in band density and iCGRP release between treatment groups and the no GDNF condition using an ANOVA with Dunnett's post-hoc test (p < 0.05). Ampersands (@) indicate statistically significant differences between the 10 ng/mL treatment condition and the condition containing the inhibitor listed below the graph using t-tests (p < 0.05). In all cases, release stimulated by capsaicin was significantly higher than basal release.

Exposure of DRG cultures to 10 ng/mL GDNF for 10 minutes doubles immunreactive phospho-Erk (p-Erk) levels compared to culture exposed to Hepes buffer alone (Figure 5A). The GDNF-induced increase in p-Erk was prevented by 10 μM PD98059 and 1.0 μM U0126, two MEK inhibitors [31-34], but not by 10 μM U0124, and inactive analog (Figure 5A and 5B). However, GDNF did not increase the amount of immunoreactive phospho-Akt (p-Akt; Figure 5A and 5C). Increases in the level of p-Erk and p-Akt are used as surrogate markers for activation of the MEK/Erk1/2 and PI-3K pathways, respectively.

MEK inhibitors also prevented the GDNF-induced increase in capsaicin-stimulated release of iCGRP from sensory neurons. As seen in Figure 5D, the enhancement in stimulated release of iCGRP induced by a 10 minute treatment with 10 ng/mL GDNF was abolished by the MEK inhibitors PD98059 (10 μM) and U0126 (1.0 μM), but not by the inactive control U0124 (1.0 μM). The PI-3K inhibitor, LY294002 (10 μM), and the inactive control for this compound, LY303511 (10 μM), did not affect the GDNF-induced enhancement in the stimulated-release of CGRP (Figure 5D). Taken together, these data indicate that GDNF-induced sensitization of sensory neurons occurs through activation of the MEK/Erk 1/2 pathway and not the PI-3K pathway.

**Figure 6:**
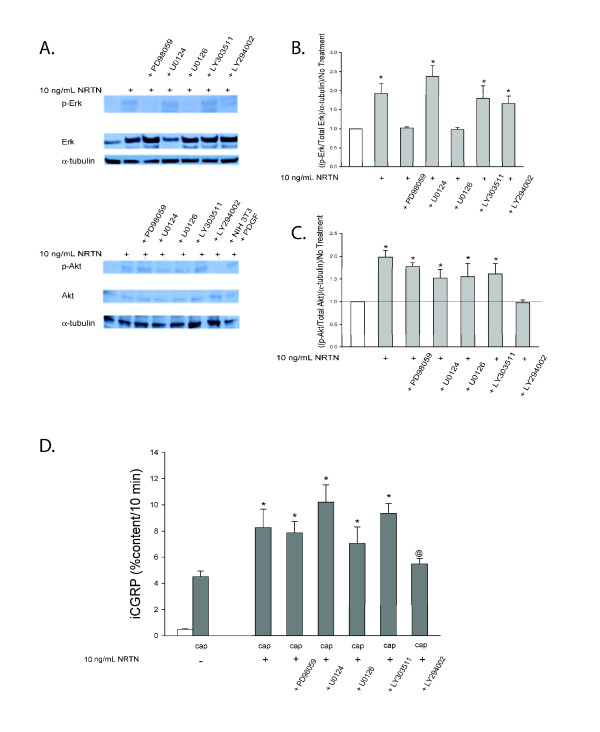
**NRTN-activates the MAPK and PI-3K pathways and induces enhancement in the stimulated-release of iCGRP through the PI-3K pathway**. A) This representative Western blot demonstrated changes in levels of p-Erk 1/2, Erk 1/2, p-Akt, Akt, and α-tubulin in DRG in response to a 10 minute incubation with 10 ng/mL NRTN and the subsequent prevention of these changes by inhibitors of the MAPK pathway (10 μM PD98059 and 1 μM U0126) and the PI-3K pathway (10 μM LY294002). B and C) Densitometric analysis of three separate Western blots like that in A probing for B) MAPK pathway components and C) PI-3K pathway components. D) Peptide release elicited by a 10 minute exposure to Hepes buffer alone (open bar) or Hepes buffer containing 50 nM capsaicin (Cap; dark bars) is expressed as mean percent total peptide content of cells in each well ± SEM (n = 12-18 wells per condition). Asterisks (*) indicate statistically significant differences in band density and iCGRP release between treatment groups and the no NRTN condition using an ANOVA with Dunnett's post-hoc test (p < 0.05). Ampersand (@) indicates statistically significant differences between the 10 ng/mL treatment condition and the condition containing the inhibitor listed below the graph using t-tests (p < 0.05). In all cases, release stimulated by capsaicin was significantly higher than basal release.

NRTN also has been shown to activate the MEK/Erk 1/2 and PI-3K pathways [35,36]. When DRG cultures were exposed to 10 ng/mL NRTN for 10 minutes, both p-Erk 1/2 and p-Akt levels were increased ~2 fold (Figure 6A). PD98059 (10 μM) and U0126 (1.0 μM) prevented the NRTN-induced increased in p-Erk, while the inactive analogue U0124 (1.0 μM) did not affect NRTN increases in p-Erk (Figure 6A and 6B). Unlike GDNF, exposure of DRG cultures to NRTN increased p-Akt levels ~2 fold, and this increase was prevented by LY294002 (10 μM) but not the inactive analog, LY303511 (10 μM; Figure 6A and 6C). Interestingly, NRTN-induced enhancement in the release of iCGRP was abolished only by LY294002, the PI-3K inhibitor (Figure 6D). Neither of the MEK/Erk 1/2 inhibitors prevented NRTN-induced sensitization despite the fact that they both inhibited NRTN-induced increases in p-Erk, nor did the MEK/Erk 1/2 inhibitors affect the activation of the PI-3K pathway. Therefore, while NRTN causes increases in both p-Erk and p-Akt, only inhibition of the PI-3K pathway prevents NRTN-induced sensitization in our model of sensory neuronal sensitization. Although NRTN activated the MEK/Erk 1/2 pathway, this pathway is not responsible for NRTN-induced increases in the stimulated release of CGRP. The ability of GDNF, but not NRTN, to cause an increase in the stimulated release of CGRP through this pathway that both GFLs activate is interesting. While the mechanisms for this signaling specificity are unknown, one potential explanation is receptor-signaling complex compartmentalization. For instance, GFRα-2 may be present primarily in a cell membrane compartment with Ret, the components of the PI-3K pathway, and the TRPV1 receptor, but not the components of the MEK/Erk 1/2 pathway. On the other hand, GFRα-1 may be present primarily in a cell membrane compartment with Ret, the components of the MEK/Erk 1/2 pathway, and the TRPV1 receptor, but not the components of the PI-3K pathway. The specific intracellular signaling cascades responsible for the effects of each of the GFLs on the stimulated release of CGRP may depend upon the components present in each particular cell membrane compartment. This observation demonstrates a dissociation of increases in levels of phosphorylated effector proteins from a functional change within the cells in culture.

**Figure 7:**
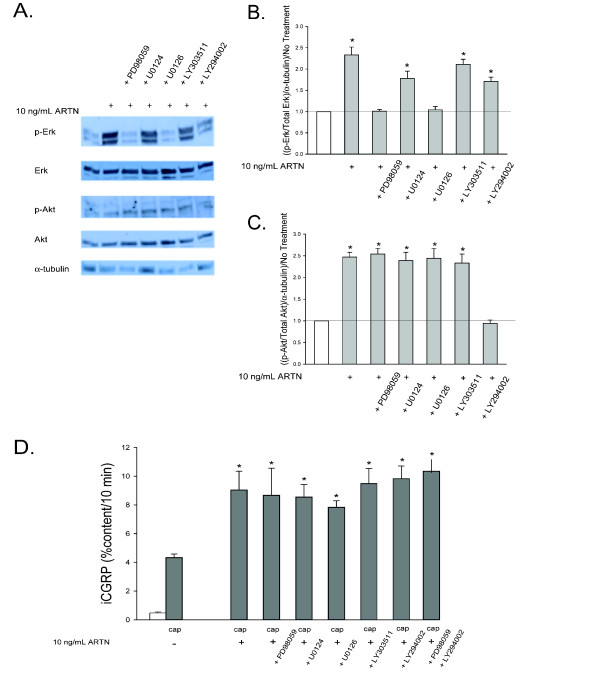
**ARTN-activates the MAPK and PI-3K pathways and induces enhancement in the stimulated-release of iCGRP through neither the MAPK nor the PI-3K pathway**. A) This representative Western blot demonstrated changes in levels of p-Erk 1/2, Erk 1/2, p-Akt, Akt, and α-tubulin in DRG in response to a 10 minute incubation with 10 ng/mL ARTN and the subsequent prevention of these changes by inhibitors of the MAPK pathway (10 μM PD98059 and 1 μM U0126) and the PI-3K pathway (10 μM LY294002). B and C) Densitometric analysis of three separate Western blots like that in A probing for B) MAPK pathway components and C) PI-3K pathway components. D) Peptide release elicited by a 10 minute exposure to Hepes buffer alone (open bar) or Hepes buffer containing 50 nM capsaicin (Cap; dark bars) is expressed as mean percent total peptide content of cells in each well ± SEM (n = 12-18 wells per condition). Asterisks (*) indicate statistically significant differences in band density and iCGRP release between treatment groups and the no ARTN condition using an ANOVA with Dunnett's post-hoc test (p < 0.05). In all cases, release stimulated by capsaicin was significantly higher than basal release.

ARTN activates a number of intracellular signaling pathways, including the MEK/Erk 1/2 and PI-3K pathways. These pathways also are associated with altered functions in sensory neurons [36-38]. A 10 minute exposure to 10 ng/mL ARTN, similar to NRTN, increased both p-Erk and p-Akt levels by 2-3 fold when compared to untreated cultures (Figure 7A, 7B and 7C). Treatment with MEK inhibitors (10 μM PD98059 and 1.0 μM U0126) prevented the ARTN-induced increases in p-Erk, while the inactive control compound, U0124 (1.0 μM), did not affect the ARTN-induced increase in p-Erk (Figure 7A and 7B). LY294002 (10 μM), the inhibitor of the PI-3K pathway, prevented the ARTN-induced increases in p-Akt, while the inactive control compound, LY303511 (10 μM), did not affect the ARTN-induced increase in p-Akt (Figure 7A and 7C). Capsaicin-stimulated release of iCGRP after a 10 min exposure to ARTN was ~2-fold higher compared to release without ARTN. The increase in stimulus-evoked release with ARTN was unaffected by inhibition of MEK by PD98059 and U0126 or inhibition of the PI-3K pathway by LY294002 (Figure 7D). This result is despite the fact that identical treatments with these inhibitors prevented ARTN-induced activation of the MAPK and PI-3K pathways, as measured by increases in p-Erk and p-Akt levels. To investigate if either pathway, MEK/Erk 1/2 or PI-3K, alone was sufficient to mediate ARTN-induced enhancement in the stimulated-release of CGRP, the MEK inhibitor PD98059 (10 μM) and the PI-3K inhibitor LY294002 (10 μM) were used in combination. When treated with both inhibitors, there was still no effect on ARTN-induced sensitization (Figure 7D), demonstrating the disconnect between increases in p-Erk and p-Akt and the functional significance of the MEK/Erk1/2 and PI-3K pathways for ARTN-induced sensory neuronal sensitization.

**Figure 8:**
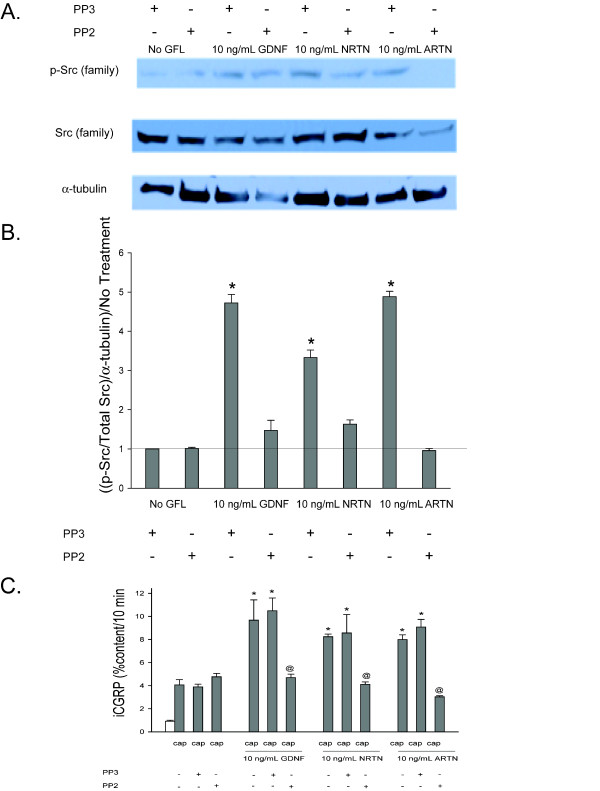
**GFL-induced enhancement in the stimulated-release of iCGRP is mediated by SFK pathway**. A) This representative Western blot demonstrated changes in levels of p-SFKs, SFK, and α-tubulin in DRG in response to a 10 minute incubation with 10 ng/mL GDNF, NRTN, and ARTN and the subsequent prevention of these changes by an inhibitor of the SFK pathway (10 μM PP2). The inactive control compound for this pathway was added as well (10 μM PP3). B) Densitometric analysis of three separate Western blots like that in A probing for SFKs. C) Peptide release elicited by a 10 minute exposure to Hepes buffer alone (open bar) or Hepes buffer containing 50 nM capsaicin (Cap; dark bars) is expressed as mean percent total peptide content of cells in each well ± SEM (n = 12-18 wells per condition). Asterisks (*) indicate statistically significant differences in band density and iCGRP release between treatment groups and the no GFL condition using an ANOVA with Dunnett's post-hoc test (p < 0.05). Ampersands (@) indicate statistically significant differences between the GFL treatment and the PP2 treated condition using a t-test (p < 0.05). In all cases, release stimulated by capsaicin was significantly higher than basal release.

**Figure 9:**
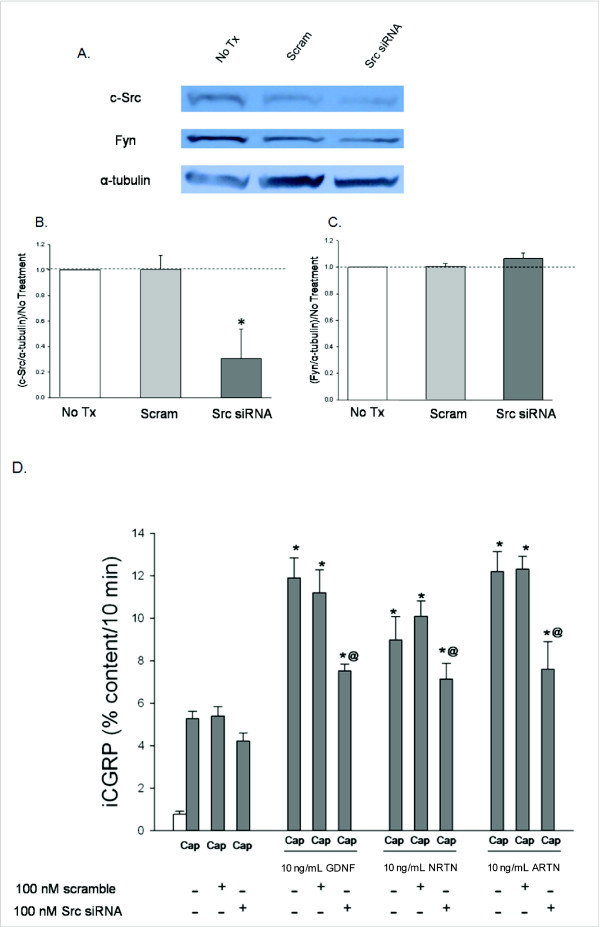
**GFL-induced enhancement in the stimulated-release of iCGRP is mediated by the Src kinase pathway**. A) This representative Western blot demonstrates reduction in levels of c-Src and no change in Fyn levels in DRG in response to Src siRNA treatment. B and C) Densitometric analysis of three separate Western blots like that in A probing for B) c-Src and C) Fyn. D) Peptide release elicited by a 10 minute exposure to Hepes buffer alone (open bar) or Hepes buffer containing 50 nM capsaicin (Cap; dark bars) is expressed as mean percent total peptide content of cells in each well ± SEM (n = 12-18 wells per condition). Asterisks (*) indicate statistically significant differences in band density and iCGRP release between treatment groups and the no GFL condition using an ANOVA with Dunnett's post-hoc test (p < 0.05). Ampersands (@) indicate statistically significant differences between the GFL treatment and the siRNA treated condition using a t-test (p < 0.05). In all cases, release stimulated by capsaicin was significantly higher than basal release.

**Figure 10:**
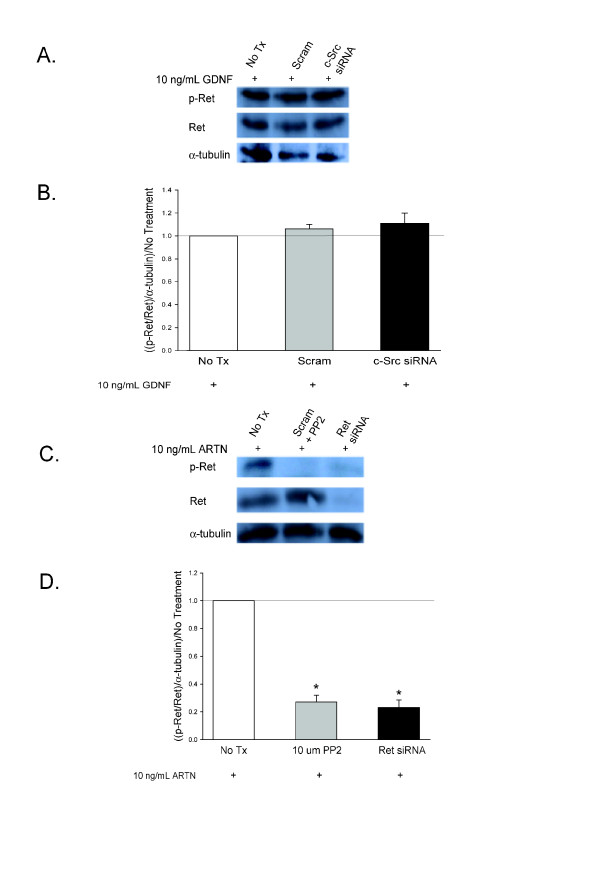
**SFK inhibition prevents Ret phosphorylation**. A) This representative Western blot demonstrates that the increase in p-Ret induced by 10 ng/mL GDNF is not prevented by either Scramble siRNA or c-Src siRNA. B) Densitometric analysis of three separate Western blots like that in A probing for p-Ret. C) This representative Western blot demonstrates that twenty minute exposure of DRG to 10 ng/mL ARTN increased p-Ret levels, and this increase was prevented by both Ret siRNA and the SFK inhibitor PP2. D) Densitometric analysis of three separate Western blots like that in A probing for p-Ret. Asterisks (*) indicate statistically significant differences between treatment conditions and the no treatment condition using an ANOVA with Dunnett's post hoc testing (p < 0.05).

**Figure 11:**
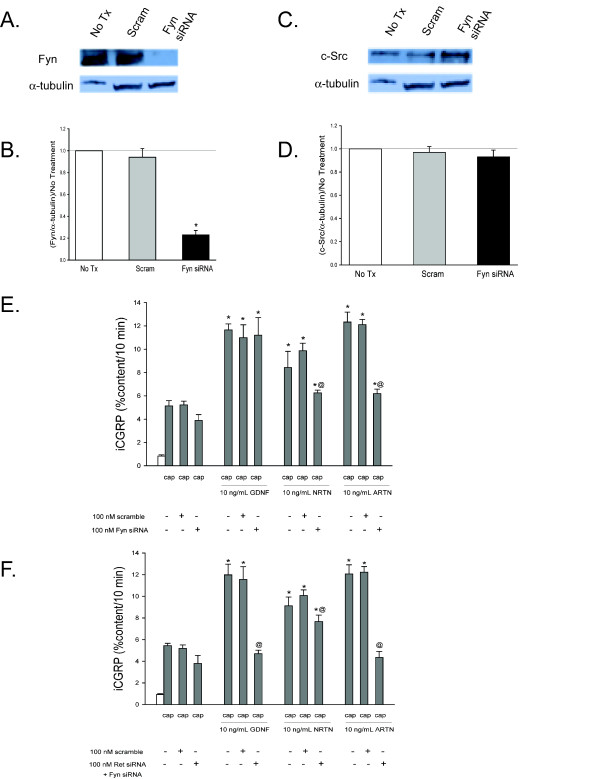
**ARTN-induced enhancement in the stimulated-release of iCGRP is mediated, in part, by Fyn-dependent pathways**. A) This representative Western blot demonstrates that exposure of DRG to Fyn siRNA decreases Fyn levels, while Scramble siRNA does not change Fyn levels. B) Densitometric analysis of three separate Western blots like that in A probing for Fyn. C) This representative Western blot demonstrates that exposure of DRG to Fyn siRNA (100 nM) and scramble siRNA (100 nM) do not change c-Src levels. D) Densitometric analysis of three separate Western blots like that in A probing for c-Src. E and F) Peptide release elicited by a 10 minute exposure to Hepes buffer alone (open bar) or Hepes buffer containing 50 nM capsaicin (Cap; dark bars) is expressed as mean percent total peptide content of cells in each well ± SEM (n = 12-18 wells per condition). Asterisks (*) indicate statistically significant differences in band density and iCGRP release between treatment groups and the no GFL condition using an ANOVA with Dunnett's post-hoc test (p < 0.05). Ampersands (@) indicate statistically significant differences between the GFL treatment and the siRNA treated condition using a t-test (p < 0.05). In all cases, release stimulated by capsaicin was significantly higher than basal release.

There is emerging evidence that the Src family kinases (SFKs), which are pathways initiated by activation of Ret, NCAM, and Integrin β-1, play an important role in sensory neuronal sensitization [39,40] and that the GFLs activate the SFKs [26,38]. To evaluate the role of SFKs in GFL-induced sensory neuronal sensitization, DRG cultures were exposed to each of the GFLs and the amount of phospho-SFKs (p-SFK), were measured with a pan-SFK antibody. Each of the GFLs increases p-SFK levels, and the pan-SFK inhibitor, PP2, at a concentration of 10 μM [41], prevented this increase (Figure 8A and 8B). The inactive analogue of PP2, PP3, did not prevent the GFL-induced increase in SFKs (Figure 8A and 8B). The pharmacological agents, PP2 and PP3, were then added to the DRG cultures to determine the role of SFKs in GFL-mediated enhancement of capsaicin stimulated release of iCGRP. PP2 abolished the sensitization of stimulus-evoked release by GDNF, NRTN, and ARTN, while the inactive control, PP3, did not affect any of the GFL-induced sensitization (Figure 8C). These experiments suggest that activation of SFKs is involved in GFL-induced sensitization. However, PP2 prevents phosphorylation of many proteins, including Src [42], the other SFKs, Fyn and Yes [26], and importantly, Ret [26]. Therefore, siRNA targeted to c-Src specifically, and not the other SFKs, was used as a tool to more specifically evaluate the role of the c-Src pathway in GFL-induced sensitization. The c-Src siRNA (100 nM) was added to the DRG cultures two days after plating and remained in the culture media for 48 hours. Figure 9A, B, and 9C show that the c-Src siRNA reduces c-Src expression levels by ~80% and does not change Fyn levels. Scramble siRNA did not affect c-Src or Fyn levels (Figure 9A, B, and 9C). Although GFL-induced enhancement in the stimulated-release of CGRP of sensory neurons was still present after treatment with c-Src siRNA, there was a reduction in the magnitude of enhancement of release of iCGRP by each of the GFLs in cultures exposed to c-Src siRNA compared to those exposed to scramble siRNA (Figure 9D). c-Src siRNA did not alter Ret expression or increases in p-Ret induced by GDNF treatment (Figure 10A and 10B), while PP2 did prevent p-Ret production induced by ARTN treatment (Figure 10C and 10D), indicating that the complete prevention of enhancement in the stimulated-release of CGRP by PP2 is not accomplished through inhibition of Src activation. The tyrosine kinase, Fyn, is a downstream effector of NCAM that is not activated by Ret [14]. To further evaluate the role of the NCAM-initiated signaling cascade in sensory neuron sensitization, Fyn expression was reduced by treatment of DRG with Fyn siRNA (100 nM). Fyn siRNA treatment reduced Fyn levels by ~80% (Figure 11A and 11B). There was no difference in Fyn levels between non-treated and scramble siRNA treated DRG (Figure 11A and 11B), and Fyn siRNA did not affect the level of the other SFK, c-Src (Figure 11C and 11D). When the DRG cultures were treated with Fyn siRNA, the GFL-induced actions on iCGRP release mimicked the results seen with NCAM siRNA. GDNF-induced enhancement in the stimulated-release of CGRP was not affected, while NRTN and ARTN-induced sensitization was still present, but the absolute amount of NRTN- and ARTN-dependent enhancement of stimulated-release of iCGRP was reduced (Figure 11E). When the DRG cultures were treated with both Ret siRNA and Fyn siRNA, the ARTN-induced enhancement in the stimulated-release of CGRP was abolished, while the NRTN-induced sensitization was still present (but the absolute amount of NRTN-induced enhancement of stimulated release of iCGRP was reduced; Figure 11F). Since only 20-50% of DRG neurons coexpress GFRα-2 and CGRP, changes in SFK phosphorylation seen in the heterogeneous DRG population may not correlate completely with changes in CGRP release in this preparation. Together, these data indicate that NCAM/Fyn signaling does play an important role in the Ret-independent component of NRTN- and ARTN-induced sensitization of sensory neurons but that NRTN-induced responses may utilize yet a third mode of activation.

**Figure 12:**
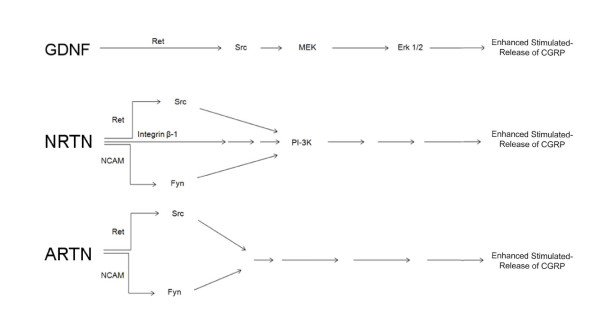
**Schematic of working model of the pathways of enhancement in the stimulated-release of CGRP by GDNF, NRTN, and ARTN**. This work was funded in part by NINDS R01 NS051668 (CMH) and a Young Investigator's Award from the Children's Tumor Foundation (BSS).

The experiments detailed above demonstrate that each of the GFLs have distinct, though overlapping, complements of signaling pathways for the induction of sensory neuronal sensitization. GDNF induces enhancement in the stimulated-release of CGRP in a Ret-dependent manner through the MEK/Erk 1/2 pathway. NRTN causes sensitization through the PI-3K pathway in both a Ret-dependent manner and a Ret-independent manner via the NCAM and Integrin β-1 receptors. ARTN induces sensitization in a Ret-dependent and Ret-independent manner, via the NCAM receptor. Downstream actions of ARTN may be mediated through PKC activation (Dr. Weiguo Zhu, personal communication). Interestingly, NRTN and ARTN induced increases in pathways unnecessary for sensitization, demonstrating a definitive dissociation of pathway activation and functional changes in the cell. The pathways of sensitization by each of the GFLs are represented schematically in Figure 12.

## Conclusions

We have demonstrated for the first time functional consequences of GFL-induced Ret-independent pathway activation in neurons. We also have demonstrated dissociation of pathway activation, as measured by increases in the level of phosphorylated effector proteins, and functional consequences of inhibition of these pathways on sensitization.

### Initiation of GFL-induced enhancement in the stimulated-release of CGRP is accomplished through several and distinct complements of cell surface receptors

Ret is the classic signaling partner of the GFL-GFRα complex, but there is increased evidence from the literature that the GFLs can signal independently of Ret in cells that lack Ret [43]. One other Ret-independent signaling mechanism for the actions GDNF is directly through the GFL-GFRα complex [44,45]. The GDNF-GFRα complex can bind to Integrin β-1 [16]. We did not demonstrate a Ret-independent component for GDNF-induced enhancement in the stimulated-release of CGRP. This could be accounted for by the use of different cell types and cell functions studied. GDNF promoted ureteric branching, but not chemotactic migration, independently of Ret [45], and embryonic substantia nigra neurons were protected from 6-OH DA damage through NCAM [14,16]. The effects of GDNF in these cells were likely due to the growth promoting effects of GDNF, distinct from sensitization. There is no evidence for NRTN-induced, Ret-independent effects in any cell type. Our demonstration of NRTN-induced, Ret-independent pathway of sensitization is novel. The NCAM-dependent actions of NRTN may be mediated by the direct binding of NRTN with NCAM, since GFRα-2 levels may be decreased in sensory neurons in culture [3]. ARTN also alters sensory neuronal sensitization through Ret-independent mechanisms [46,47]. The normal electrophysiological functions of injured C-fibers are recovered by exposure to ARTN. This recovery occurs for C-fibers that express GFRα-3 but not Ret, demonstrating these effects are Ret-independent [46]. Together, this suggests a role for Ret-independent actions of NRTN and ARTN in sensory neurons.

### GDNF-induced enhancement in the stimulated-release of CGRP is mediated by the MAPK/Erk 1/2 pathway

GDNF activates the MAPK/Erk 1/2, the PI-3K, and the Src kinase pathways [5,48]. GDNF robustly activated the MAPK/Erk 1/2 and Src pathways, but not the PI-3K pathway in our DRG cultures. MAPK/Erk 1/2 pathway inhibitors, SFK inhibitors, and Src siRNA prevented GDNF-induced enhancement in the stimulated-release of CGRP, while inhibition of the PI-3K pathway did not. Bron et al. observed PI-3K activation with exposure to GDNF [48]. However, in this study the DRG were exposed to high concentrations of GDNF (50 ng/mL and higher), which could account for the PI-3K pathway activation. Activation of PI-3K also could have occurred through non-specific effects of GDNF through non-specific binding to the GFRα-2 receptor [49-51] at the higher concentrations used.

There is additional evidence that GDNF can activate SFKs in a Ret-independent manner [5,52] in DRG from Ret deficient mice and two neuronal cell lines that lack Ret expression (NIH3T3 and SHEP neurobalstoma), stably transfected with GFRα-1. When Ret was inhibited using a specific siRNA, the GDNF-induced sensitization was abolished. The differences between previous reports of GDNF-induced, Ret-independent actions and the data presented here could be the result of the different developmental stage and type of cells. Embryonic neurons and cell lines may be primarily responding to growth promoting actions of GDNF, which may use different complements of signaling pathways and cell surface receptors, than adult primary DRG preparations.

### NRTN-induced enhancement in the stimulated-release of CGRP is mediated by the PI-3K pathway

There is evidence for NRTN activation of MAPK, PI-3K, and SFK pathways [35-37]. NRTN robustly activated all three of these pathways. However, NRTN-induced sensory neuronal sensitization was prevented by inhibition of the PI-3K and SFK pathways, but not the MAPK pathway. The downstream effector of NRTN-induced sensitization was in all cases PI-3K.

### ARTN-induced enhancement in the stimulated-release of CGRP is mediated by neither the MAPK/Erk 1/2 pathway nor the PI-3K pathway

ARTN also activates the MAPK, PI-3K, and SFK pathways [35-37]. Inhibition of any of these pathways could prevent ARTN-induced enhancement in the stimulated-release of CGRP. However, only Src inhibition was able to reduce the amount of ARTN-induced sensitization. Inhibitors of the MAPK/Erk 1/2 or the PI-3K pathways did not prevent the ARTN-induced sensitization even though they effectively reduced the levels of p-Erk and p-Akt. Either of these pathways may be sufficient, but neither necessary, for ARTN-induced sensitization. There is evidence for the need for both MAPK and PI-3K activation for neuronal protection by GDNF [53]. Addition of GDNF to B92 glial cells prevented damage of these cells by high concentrations of ethanol through the MAPK and PI-3K pathways [53]. Inhibition of either pathway individually did not reverse the effects of GDNF. The use of one inhibitor of the MAPK/Erk 1/2 and one inhibitor of the PI-3K pathway in combination did not affect the enhancement in stimulated-release of iCGRP induced by ARTN. These data demonstrate that Src is a likely pathway important in ARTN-induced sensitization, but that neither MAPK nor PI-3K is necessary for this sensitization. While previous studies identified either or both of these pathways as important in alteration of neuronal function by ARTN [36-38], these studies were conducted on motor neurons or glial cells, not adult sensory neurons. These differences in cell type could explain the different pathways activated.

### Dissociation of intracellular signaling pathway activation and induction of enhancement in the stimulated-release of CGRP by these pathways

The work presented here demonstrates the dissociation of pathway activation and function. NRTN and ARTN induced increases in phosphorylated effector proteins for the MAPK and PI-3K pathways, but these pathways were unnecessary for NRTN- or ARTN-induced enhancement in the stimulated-release of CGRP. NRTN increases both p-Erk and p-Akt levels, but only inhibition of the PI-3K pathway prevented NRTN-induced sensitization. ARTN increased the level of p-Erk and p-Akt, however, inhibition of either of these pathways or both pathways simultaneously did not prevent sensitization. Previous work demonstrating the role of the MEK/Erk1/2 and PI-3K pathways in sensory neuronal sensitization has been predicated on the fact that increases in p-Erk and p-Akt are adequate surrogate measures for function changes.

### Possible physiologic purpose for different complements of receptors and intracellular signaling pathways for enhancement in the stimulated-release of CGRP by each of the GFLs

Although GDNF, NRTN and ARTN were originally thought to mediate all of their actions through the Ret receptor and similar sets of intracellular signaling pathways, we have demonstrated that each of these GFLs uses distinct complements of signaling pathways to sensitize sensory neurons. There are three possible explanations for these differences. First, each of the GFLs is modulating the responses of sensory neurons innervating different tissues. The DRG is a heterogenous population of neurons; in particular there are sensory neurons that innervate the skin, the viscera, and the musculoskeletal system. There is evidence that each of the GFLs has specific and/or preferential populations of sensory neurons on which they modulate responses [54-58]. It is possible that each of the subtypes of primary afferents has distinct sets and abundances of receptors (Ret, NCAM, and Integrin β-1) and signaling pathways available for the action of sensitization. Muscle afferents and motor neurons, under certain circumstances, may express only Ret and GFRα-1 receptors and preferentially use the c-Src kinase/MAPK/Erk 1/2 pathway for sensitization, while visceral afferents may express GFRα-2, Ret, NCAM, and Integrin β-1 receptors and preferentially use the SFK and Syk kinase signaling cascades through the PI-3K pathway for sensitization. ARTN responsive neurons may express only Ret, NCAM, and GFRα-3 receptors and preferentially use the Fyn kinase and c- Src kinase signaling cascade for sensitization. Second, each of the specific GFRα receptors may localize to different portions of the cell membrane where different complements of receptors and pathways are present. When the GFL-GFRα complex binds its GFL, this complex recruits Ret into lipid rafts and initiates signaling [59]. Inside lipid rafts, Ret signals through SHC and Grb2 [60]. Outside lipid rafts, Ret signals through FSR2 [60]. Each of the GFLs could be activating different receptors and signaling pathways based on compartmentalization of these receptors and pathways with individual GFRα receptors. Finally, each of the GFLs may cause different structural changes in their specific GFRα receptor subtype that allow different interactions with Ret, NCAM, and Integrin β-1. It has been shown that when the ARTN-GFRα-3 complex translocates to Ret, it activates the MAPK pathway more slowly and less robustly than when the GDNF-GFRα-1 complex translocates to this receptor [13]. This may be because different tyrosine residues are available depending on the Ret configuration. This could partially explain the differential complements of pathways used by each of the GFLs to accomplish their sensory neuronal sensitization.

GFLs can induce sensitization of sensory neurons in a Ret-independent manner. Additionally, it is clear that increases in phosphorylated effector proteins do not establish a causal role for that effector system in functional endpoints. Knowledge of the signaling pathways available for use by the GFLs may be useful in better understanding and control of the pathophysiological role that the GFLs play in cellular processes including inflammation and pain.

## Methods

### Materials

The mice used for all experiments, C57BL/6 mice, were purchased from Harlan Laboratories (Indianapolis, IN) and/or bred and housed in the Indiana University Laboratory Animal Research Center (LARC). All mice were adults, between three and six months in age. All experiments were performed in accordance with National Institutes of Health Guide for Care and Use of Laboratory Animals. All procedures were reviewed and approved by the Indiana University School of Medicine Institutional Animal Care and Use Committee. Capsaicin was purchased from Sigma Chemical Company (St. Louis, MO) and was first dissolved in 1-methyl,2-pyrrolidinone (Aldrich Chemical Co., Milwaukee, WI) to a concentration of 10 mM. It was then serially diluted to a concentration of 50-500 nM in the appropriate release buffer as noted below. Horse serum, F-12 medium, L-glutamine, and penicillin/streptomycin were purchased from Invitrogen (Carlsbad, CA, USA). NGF was purchased from Harlan Bioproducts for Science, Inc. (Indianapolis, IN, USA). Collagenase, poly-D-lysine, laminin, 5-fluoro-2-deoxyuridine, uridine and standard laboratory chemicals were from Sigma (St. Louis, MO, USA). Antibody to calcitonin gene-related peptide (CGRP) was generously provided by Michael R. Vasko (Indiana University School of Medicine, Indianapolis, IN, USA and originally produced by Michael J. Iadarola, NIH). The GFLs were purchased from Peprotech (Rocky Hills, NJ). The siRNA constructs were purchased from Santa Cruz Biotechnology (Santa Cruz, CA, USA) and were all pooled siRNAs except for the Src and scramble siRNAs (not pooled siRNAs) which were developed by Eric L. Thompson in Dr. Michael L. Vasko's laboratory (Indiana Univ. School of Medicine) and created by Dharmacon, Inc (Lafayette, CO, USA). An siRNA designed as a scramble for APE1, a multifunctional DNA repair enzyme, was used as an siRNA transfection control, as this control has been used in several sets of experiments in the Hingtgen, Vasko, and Kelley laboratories and has not shown activity against any protein of interest in any of these studies [61,62]. The Ret protein control, isolated from mice, was purchased from Santa Cruz Biotechnology. The sources, dilutions, and place of purchase of each of the primary antibodies are listed below. They are Ret: rabbit polyclonal primary antibody, 1:500 titer, Santa Cruz Biotechnology; p-Ret: rabbit polyclonal primary antibody, 1:300 titer, Santa Cruz Biotechnology; α-tubulin: mouse monoclonal antibody, 1:1,000 titer, Sigma; NCAM: rabbit polyclonal primary antibody, 1:500 titer, Santa Cruz Biotechnology; Integrin β-1: mouse monoclonal primary antibody, 1:300 titer, Santa Cruz Biotechnology; p-Erk 1/2: mouse monoclonal primary antibody, 1:500 titer, Cell Signaling Technologies; Erk 1/2: rabbit polyclonal primary antibody, 1:900 titer, Cell Signaling Technologies; p-Akt: mouse monoclonal primary antibody, 1:500 titer, Cell Signaling Technologies; Akt: rabbit polyclonal primary antibody, 1:800 titer, Cell Signaling Technologies; p-SFKs: rabbit polyclonal primary antibody, 1:500 titer, Cell Signaling Technologies; SFK: rabbit polyclonal primary antibody, 1:500 titer, Cell Signaling Technologies; c-Src: mouse monoclonal primary antibody, 1:500 titer, Santa Cruz Biotechnology; Fyn: mouse monoclonal primary antibody, 1:300 titer, Santa Cruz Biotechnology. The sources, dilutions, and place of purchase of each of the primary antibodies are listed below. They are goat anti-rabbit secondary antibody, 1:25,000 titer, Jackson Laboratories and goat anti-mouse secondary antibody, 1:10,000 titer, Jackson Laboratories.

### Preparation of dorsal root ganglia (DRG) cultures

Dorsal root ganglia (DRG) from adult mice were used to establish sensory neuronal cultures. Briefly, the DRG were removed from adult mice and prepared as previously published [4]. Cells were plated in wells of 24-well Falcon culture dishes coated with poly-D-lysine and laminin at a density of 30,000-50,000 cells/well. Cultures were maintained at 37°C in a 5% CO_2 _atmosphere in F12 media supplemented with 2 mM glutamine, 50 μg/mL penicillin and streptomycin, 10% heat-inactivated horse serum and mitotic inhibitors (50 μM 5-fluoro-2-deoxyuridine and 150 μM uridine). NGF, at a concentration of 30 ng/mL, was added to this media. Growth medium was changed every 2-3 days, and the added NGF removed 48 hrs prior to all experiments.

### Stimulated-Release of iCGRP

Measurement of stimulus-evoked release and content of immunoreactive CGRP (iCGRP) from isolated sensory neurons was accomplished as previously published [4]. After 5-7 days in culture, culture media was removed from the sensory neurons in culture and the basal or resting release of iCGRP measured from cells incubated for 10 minutes in HEPES buffer consisting of (in mM): 25 HEPES, 135 NaCl, 3.5 KCl, 2.5 CaCl2, 1 MgCl_2_, 3.3 dextrose, and 0.1% (w/v) bovine serum albumin, pH 7.4, and maintained at 37°C. The cells were incubated in HEPES buffer containing stimulus (capsaicin, 50 nM) for 10 minutes, and then incubated again with HEPES buffer alone to reestablish resting release levels. The amount of iCGRP released in each incubation was measured by radioimmunoassay (RIA). The minimum amount of iCGRP detected by the RIA is 5 fmol with a 95% confidence interval [63]. After the release protocol, the remaining peptide content in each well was determined by exposing the cells to 2 N acetic acid for 10 minutes. Aliquots of this incubation were diluted in HEPES and iCGRP was determined by RIA. The total content of iCGRP in the DRG cultures was not altered by any of the siRNA or pharmacological treatments (data not shown). The release of iCGRP during the 10 min incubation period is expressed as percent of the total content. GFLs and pharmacological inhibitors were added in the basal incubation period (10 minutes) and in the stimulated incubation period (10 additional minutes) for a total exposure time of 20 min. A minimum of three different preparations were used for each condition, including growth factor application and pharmacological inhibitor application.

### Treatment of DRG with siRNA and/or Pharmacological Inhibitors

When using siRNA to inhibit specific protein production, these molecules were added two days after DRGs were plated. Metafectine Pro (Biontex Laboratories, Martinsried, Planegg, Germany), the transfection agent, was diluted to a titer of 1:250 in each well in Optimem reduced serum media (Invitrogen, Carlsbad, CA). The siRNA molecules were also diluted in Optimem. The Metafectine and siRNA dilutions were allowed to sit at room temperature for two minutes then mixed at a 1:1 ratio and allowed to incubate at room temperature for 20 minutes. The mixture was added to each well so that the final concentration of the siRNA was 100 nM. The following day, F12 media containing NGF and normocin was added to the wells to a final volume 1.0 mL. Twenty four hours later, all the media was removed from the wells and 500 μl of normal growth media (F12 media supplemented with F12 media supplemented with glutamine, penicillin and streptomycin, horse serum and mitotic inhibitors as indicated above) was added. Cells were maintained in F12 media without supplemental NGF for the 48 hours prior to use in experiments.

### Isolation of protein samples from sensory neurons in culture and Western blotting

Sensory neurons from DRG from adult mice plated on 12 well Falcon plates were maintained in culture for 5-7 days. Cells were then washed with Hepes buffer and treatments added as indicated below. After treatment, the cells were washed once with PBS, then 500 μl of PBS added to each well. The wells were then scraped and the cells transferred in solution to ependorf tubes. The tubes were centrifuged at 16,000 rpm for 20 minutes. The supernatant was removed and the remaining pellet was either placed on dry ice and transferred immediately to a freezer at -80°C or protein content quantified immediately.

For protein quantification, DRG pellets were resuspended in 50 μl of general lysis buffer (1mM Na pyrophosphate, 50 mM Hepes, 1% Triton X-100, 50 mM NaCl, 50 mM NaF, 5 mM EDTA, and 1 mM Na orthovanadate) supplemented with proteinase inhibitor mixture (aprotinin, leupeptin, pepstatin, PMSF; Calbiochem, Sand Diego, CA, USA). The resuspended protein was incubated for 15 minutes on ice with frequent vortexing. The suspension was Sonicated 3 times for 10 seconds each at 45 watts. The suspension was then centrifuged at 4,000g for 2 minutes. The supernatant was removed and stored at -20°C. The protein was quantified using a BCA Protein Assay Kit (Thermo Scientific, Rockford, IL, USA) and read on a Wallac plate reader at 595 nm for 1.0 s.

A total of 40 μg of the protein samples were mixed with loading buffer (Ambion, Austin, TX, USA) containing β-mercaptaethanol to a final volume of 60 μl and denatured at 70°C foir 10 minutes. The samples were then incubated at room temperature for 15 minutes and loaded into wells of precast 10% SDS-PAGE gels (Biorad, Philadelphia, PA, USA) containing 10 lanes. The samples were run on the gels, which were connected to a Biorad power source, for 2 hours at 115 mV at room temperature. While the gel was running, filter papers (Biorad, Philadelphia, PA, USA), fiber pads (Biorad, Philadelphia, PA, USA), and PVDF transfer membranes (Millipore, Darwinweg, the Netherlands) were soaked in 1X transfer buffer (25 mM Tris at pH 7.5; 192 mM Glycine; 5-20% methanol). Prior to soaking in transfer buffer, the PVDF membranes were soaked in 100% methanol for 1 min and washed extensively with ddH_2_0. SDS-PAGE gels were placed on transfer membranes within a transfer cartridge and transferred in a Biorad system at 100 mV for 1 hour at room temperature with an ice pack in the apparatus.

After transfer, the membranes were removed from the apparatus and placed in 10% powered skim milk (EMD, Gibbstown, NJ, USA) in 1X TBS (20 mM Tris PH 7.5; 150 mM NaCl as a blocking solution for 1 hour at room temperature. Then, the blocking solution was discarded and the membranes placed in 5% milk in TBST (TBS containing 0.1% Tween-20) containing primary antibodies at concentrations of 1:200 to 1:1,000. The membranes were incubated in this solution overnight at 4°C. Several short washings and three 10 minute washings were accomplished with TBST after the overnight incubation. Secondary antibody, at concentrations from 1:4,000 to 1:25,000, in 5% milk in TBST was applied to the membrane for 1 hour at room temperature. A similar set of washings was done after the secondary antibody exposure, then the membranes were blotted dry and placed in the combination of solutions for enhanced chemiluminescence (ECL; Thermo Scientific, Rockford, IL, USA) for 3 minutes. The membranes were placed in clear plastic sheets and inserted into X-ray cartridges (Soyee Products, New York City, NY, USA). Once in complete darkness, X-ray films (Midwest Scientific, St. Louis, MO, USA) were placed inside the cartridge and exposed for 5 seconds, 30 seconds, 1 minute, 5 minutes, and 15 minutes. Films were first placed in Kodak/GBX developer (Thermo Fisher Scientific, Pittsburgh, PA, USA) for 30 seconds per side, then washed in cold water for 1 minute per side. The films were fixed in Kodak/GBX fixing solution (Thermo Fisher Scientific, Pittsburgh, PA, USA) for 30 seconds per side, washed again, and allowed to dry for 2 hours.

The dried films were scanned as JPEG files and densitometric measurements made with Un-Scan It (Orem, UT, USA). Immunoreactive bands of interest were normalized to α-tubulin bands.

### Statistical Analyses

Results, represented as percent total content of iCGRP, are expressed as the mean ± standard error of the mean (SEM). All differences were compared with one-way analyses of variance (ANOVAs) and Dunnett's post hoc analysis or Student t-tests, as indicated. A p value of <0.05 was used to indicate statistical significance.

## List of Abbreviation

ARTN: Artemin; Cap: Capsaicin; CGRP: Calcitonin gene-related peptide; DRG: Dorsal root ganglia; Erk 1/2: Extracellular signal-related kinase 1/2; GDNF: Glial cell line-derived neurotrophic factor; GFL: Glial cell line-derived neurotrophic factor family ligands; GFRα: GDNF family receptor alpha; KCl: Potassium chloride; IB4: Isolectin B4; LPS: Lipopolysaccharide; MAPK: Mitogen-activated protein kinase; NCAM: Neural cell adhesion molecule; NGF: Nerve growth factor; NRTN: Neurturin; PI-3K: Phosphoinositide-3 Kinase; PSPN: Persephin; RIA: Radioimmunoassay; SEM: Stardard error of the mean; TRPV1: Transient receptor potential vanilloid 1.

## Competing interests

The authors declare that they have no competing interests.

## Authors' contributions

BSS conceived of the study, participated in its design, sacrificed the mice, established and maintained the DRG cultures, conducted the iCGRP release studies, conducted the Western blot imaging, analyzed the data, and was the primary author of the manuscript. SR maintained the mouse colony, assisted in harvesting the DRG from the mice, assisted in sacrificing the mice, and assisted in maintaining the DRG cultures. SKP assisted in harvesting the DRG from the mice, assisted in sacrificing the mice, and assisted in maintaining the DRG cultures. RMM assisted in harvesting the DRG from the mice, assisted in sacrificing the mice, and assisted in maintaining the DRG cultures. CMH provided the laboratory and equipment/supplies for the studies, aided in developing the design of the studies, and provided editorial assistance. Additionally, the rationale for these studies were based upon an extension of previously conducted and proposed studies by CMH. All authors read and approved the final manuscript.
